# Lumbar Microdiscectomy: A Rare Case of a Duplicate L5 Exiting Nerve Root

**DOI:** 10.7759/cureus.92469

**Published:** 2025-09-16

**Authors:** Sahil Garg, Sean M Muir, Arianna K Gill, Komi E Afetse, Sanjitpal S Gill

**Affiliations:** 1 Orthopaedic Surgery, Steadman Philippon Research Institute, Vail, USA; 2 Medicine, University of South Carolina School of Medicine Greenville, Greenville, USA; 3 Internal Medicine, OhioHealth Riverside Methodist Hospital, Columbus, USA; 4 Orthopedics, Steadman Philippon Research Institute, Vail, USA; 5 Orthopaedic Research, Steadman Philippon Research Institute, Vail, USA; 6 Orthopaedic Spine Surgery, The Steadman Clinic, Vail, USA

**Keywords:** anomalous nerve, case report, dual exiting nerve root, microdiscectomy, nerve root classifications, surgical complications

## Abstract

The microdiscectomy procedure has been regarded as the preferred method for the treatment of lumbar disc herniation (LDH). Intraoperatively, the procedure may be further complicated due to underlying anatomical variations in the lumbar spine or nerve root variations. Nerve root anomalies (NRAs), in particular, are relatively underdiscussed within the literature. Despite the rarity of these variations to the exiting nerve root, the increased surgical risk that an NRA may add warrants the need for increased awareness and associated complication mitigation. This case describes a rare instance of a symptomatic duplicate L5 nerve root discovered preoperatively and highlights the steps taken to minimize iatrogenic risk. This study is designed as a retrospective case report with formal informed consent provided by the patient. A thorough chart review was performed for this subject, including all prior medical records, imaging studies, imaging reports, operative notes, and communication records. This patient’s preoperative lumbar MRI demonstrated severe left foraminal narrowing at L5-S1 with disc encroachment upon the exiting L5 nerve root and traversing S1 nerve root. The patient was described as having a type 1a NRA. Meticulous consideration was made intraoperatively with no known sustained iatrogenic injury. Preoperative awareness of NRAs, careful dissection, and thorough intraoperative visualization of neural structures are paramount to minimizing the risks of surgical complications. Surgeons should be aware of NRA classification systems and have a surgical plan in place to mitigate risk. Improved awareness may increase the likelihood of preoperative diagnosis and prevent iatrogenic injury.

## Introduction

Surgical intervention of the lumbar spine is commonly indicated in patients with underlying neurological weakness. Commonly cited causes of neurological weakness that may improve from surgical intervention include central and foraminal stenosis. These pathologies occur more frequently in the cervical and lumbar spine. In the lumbar spine, levels L4-5 and L5-S1 are the most frequently compromised intervertebral discs associated with the aforementioned pathologies [1].

The L5/S1 nerve root emerges from the spinal cord through the intervertebral foramen formed between the fifth lumbar vertebra (L5) and the first sacral vertebra (S1), which are part of the lumbosacral plexus. The L5 nerve root crosses the L4/5-disc space centrally or traverses and exits the spinal canal beneath the L5 pedicle. It then crosses the L5/S1 disc space at its lateral margin. It consists of a bundle of nerve fibers that branch out to provide motor signals and sensory input to different regions, including the lower back, buttocks, hips, and legs. Disruption or compression of the L5/S1 nerve root due to pathologies such as paracentral disc herniation, spinal stenosis, or sciatica may lead to significant physical and mental disability in patients. Disc herniations may occur in both traumatic and atraumatic degenerative pathologies. In this pathology, the intervertebral disc may extrude into the central or foraminal spaces of the spine and compress such neural elements. Disability is often associated with symptomatic lower back pain, lower extremity weakness, numbness, or tingling.

Sciatica is a broad clinical diagnosis associated with the compression of one or more of the nerve roots that define the sciatic nerve (L4-S3). Sciatica and symptoms of numbness and tingling in the lower extremities are most commonly due to a herniated disc in one of the lower two lumbar discs. For clarity, this is either between the fourth and the fifth lumbar vertebrae (L4-L5) or between the fifth lumbar and the first sacral vertebra (L5-S1). The most frequently affected root is L5 just before it exits the dural sac, adjacent to the L4-L5 disc, or, less commonly, it can be compressed between the L4 and L5 vertebrae in the L5-S1 intervertebral foramen, at the exit of the spinal canal, in the intervertebral foramen between L4 and L5 [2].

Lumbar disc herniations (LDH) are a frequent and early marker of degeneration in the lumbar spine. The reported occurrence of LDH is approximately 2%-3% with a prevalence of around 12% [3]. In the majority of cases, the symptoms originating from a herniated disc are successfully managed without surgery. However, for patients who do not experience relief from conservative approaches, such as physical therapy, rest, or transforaminal epidural steroid injections, surgical interventions may be considered as a potential treatment option [4,5]. Surgery for LDH accounts for the most common indication for performing spinal surgery [6]. Due to the high incidence of symptomatic LDHs in the general population and evolving nature of surgical techniques, the mainstay of operative LDH treatment is trending towards more minimally invasive approaches.

To this date, the microdiscectomy procedure has been widely regarded as the preferred method for treatment of LDH due to a series of proposed advantages over more traditional techniques. These potential benefits over the standard open discectomy include smaller incisions, decreased risk of iatrogenic instability, decreased blood loss, and potentially enhanced visualization through the use of an endoscope and other emerging technologies [7]. In the properly selected patient with underlying neurological weakness, the microdiscectomy may be considered a safe and efficacious procedure [8]. Despite the proposed benefits of this minimally invasive procedure, there are a variety of complications that both surgeons and patients should be aware of. Such complications may include hematoma, surgical site infection (SSI), durotomy, paralysis, permanent disability, and death [9].

The procedure can further be complicated due to underlying anatomical variations in the lumbar spine or nerve root variations. Nerve root anomalies (NRAs) can present in different ways, including variations in the number, position, or branching pattern of the roots. Some common types of NRA are conjoined nerve roots, accessory or duplicated nerve roots, nerve root fenestration, transverse or high division of nerve roots [10,11].

Lumbosacral NRAs are very rare and sometimes identified incidentally during diagnostic imaging studies, such as MRI or CT scans. In most cases, these anomalies are asymptomatic and do not require specific treatment. However, in cases of surgically correctable pathologies, such as lumbar foraminal or lumbar central stenosis, they may be a cause for concern. In cases where NRAs are not adequately visualized on preoperative imaging, they increase the overall surgical risk profile for patients. This case describes a rare instance of a symptomatic duplicate L5 nerve root discovered intraoperatively during an L5-S1 microdiscectomy and highlights the technical approach taken to minimize intraoperative risk. This paper also describes the process that was utilized to adequately decompress the anomalous neurological element safely, with patient-reported improvement of underlying neurological weakness postoperatively.

## Case presentation

A 50-year-old female patient presented to our Orthopedic Spine practice for evaluation and treatment of axial back pain with associated left buttock and left posterolateral radicular leg pain. The patient was involved in a motor vehicle accident (MVA) 18 months prior to her clinical visit. Her vehicle was rear-ended, and there were no deaths at the scene. She developed left-sided radicular symptoms four days after the MVA. She tried a course of physical therapy for several months and lumbar steroid injections, with only mild transient relief of her symptoms. The patient rated her pain as 7 out of 10 in severity and stated that her symptoms cause a significant amount of disability in her day-to-day activities. Patient denied incontinence of bowel or bladder and had no constitutional symptoms. This patient did have a family history of adolescent idiopathic scoliosis and a history of mild degenerative scoliosis, which has been mostly asymptomatic.

During the clinical exam, the patient was found to have weakness of her left extensor hallucis longus (EHL), tibialis anterior, and peroneal muscle groups, scored as a 4 out of 5 using the standardized Oxford strength measurement scale. All other lower extremity muscle groups were measured as 5 out of 5 strength. She exhibited a 3+ patellar reflex on the left, 2+ patellar reflex on the right, 2+ bilateral Achilles reflexes, and negative bilateral clonus. The patient had a positive left-sided straight leg raise and a negative right-sided straight leg raise. The patient’s clinical vignette and findings indicated a left-sided LDH. These findings were also supported using a lumbar MRI scan. Relevant imaging findings included severe left foraminal narrowing at L5-S1 with encroachment of a medium-sized paracentral disc herniation upon the exiting L5 nerve root and traversing S1 nerve root.

Due to the underlying neurological weakness, positive clinical findings correlated with MRI, and an elective decision to proceed with surgery, this patient was selected as a candidate for a left L5-S1 decompression and partial discectomy.

During independent preoperative lumbar MRI film review by the surgical team, it was discovered that this patient had a duplicate L5 exiting nerve root in the left neural foramen between the L5 and S1 vertebrates. This was confirmed intraoperatively through visual inspection during the decompression. This anatomical variation was not described in any radiological reports by a radiologist. Meticulous consideration was made during the procedure with no known iatrogenic injury to either of the L5 neural elements. These specific details will be described in the discussion section of this report.

Postoperatively, this patient suffered from no immediate complications with immediate improvement of their left-sided lower extremity weakness in the postanesthesia care unit (PACU). At the patient’s two-week post-operative follow-up visit, she reported improvement in her underlying neurological weakness, baseline pain scores, and left-sided radicular symptoms.

## Discussion

NRAs rarely occur and are sometimes diagnosed preoperatively or as incidental findings on MRI. While described within the literature, there remains no universally accepted classification system for NRAs. This is likely due to their rarity and anatomical variance. Missed conjoined nerve roots (CNRs) present with a significant risk of iatrogenic nerve injury during surgery. Brown et al. [12] highlighted the potential differences in presentations between lumbar NRAs associated with a disc herniation on a lumbar MRI [12,13]. They also reported that potential indistinction of NRAs preoperatively has led to an increase in missed diagnoses of CNRs.

There are two predominant classification systems, Cannon and Neidre & MacNab [14]. CNRs are a rare condition that occurs when two spinal nerves traverse an aberrant embryological pathway, resulting in an anomalous communication between them. CNRs can exit above, at the same level, or below the level at which they are traditionally identified. Many CNRs have been cited as asymptomatic and are typically not discovered until common lumbar pathologies incite back pain.

Cannon et al. were the first to propose a CNR classification in 1962 [15,16]. This classification system primarily focused on whether the NRA was intradural or extradural and utilized the categories of type 1, type 2, and type 3. Any deformity with intradural spacing was considered type 1. Type 2 is defined using this system by the presence of extradural anastomotic deformities. Finally, type 3 deformities specifically consist of extradural spacing deformities [15]. While not popularized for the sake of chronological completeness, Postacchini et al. in 1982, reclassified intradural spacing, extradural spacing, and extradural anastomotic deformities as type 4, types 1-3, and type 5, respectively [11,16].

In 1983, Neidre and McNab assigned a new classification system to NRAs [14]. After a thorough literature review, this classification system was found to be the most widely accepted and published [14,16,17]. The Neidre and McNab classification expanded on the Cannon et al. system [15]. Type 1a classifications include intradural CNR anomalies where two nerve roots share a common dural sheath. Type 1b classifications are a similar variant where the nerve roots are almost connected, resulting in a nerve root that exists at right angles to the dural sheath, such as a cervical nerve root. Type 2 anomalies are extradural deformities where two nerve roots exit through a single foramen. Type 2 anomalies are subclassified into type 2a and type 2b. A type 2a NRA is where one of the existing nerve root canals is left unoccupied. In a type 2b classification, both neural foramina have an occupying nerve root, and one foramen has an additional nerve root. Type 3 anomalies are anastomotic in nature and can be described as either intradural or extradural. These nerves are anomalous such that they form a loop and either result in two nerve roots exiting a single foramen or no nerve roots exiting a foramen.

Other proposed classifications after Neidre and McNab in 1983 are Kadish and Simmons in 1984, Chotigavanich and Sawangnatra in 1992, and Haviarová et al. in 2020 [11,16,18,19]. These classifications highlight similar anomalies presented by Neidre and McNab [14]. These systems emphasize intradural, extradural, anastomotic, non-anastomotic, and spacing deformity characteristics of an aberrant root, traversing root, conjoined root, or caudal root [16,18,19].

**Figure 1:**
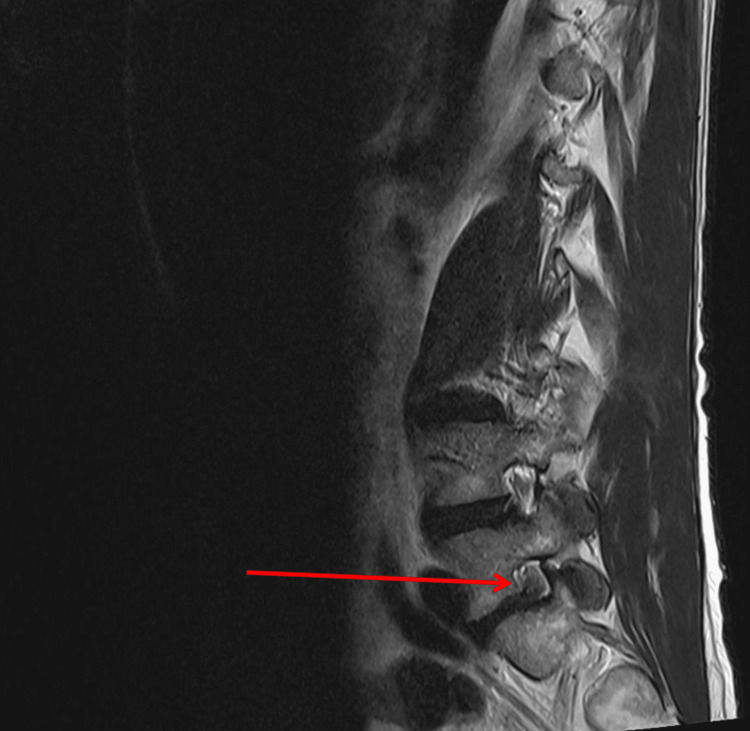
T2-weighted left parasagittal MRI of the lumbar spine. The red arrow depicts a duplicate left L5 exiting nerve root.

**Figure 2:**
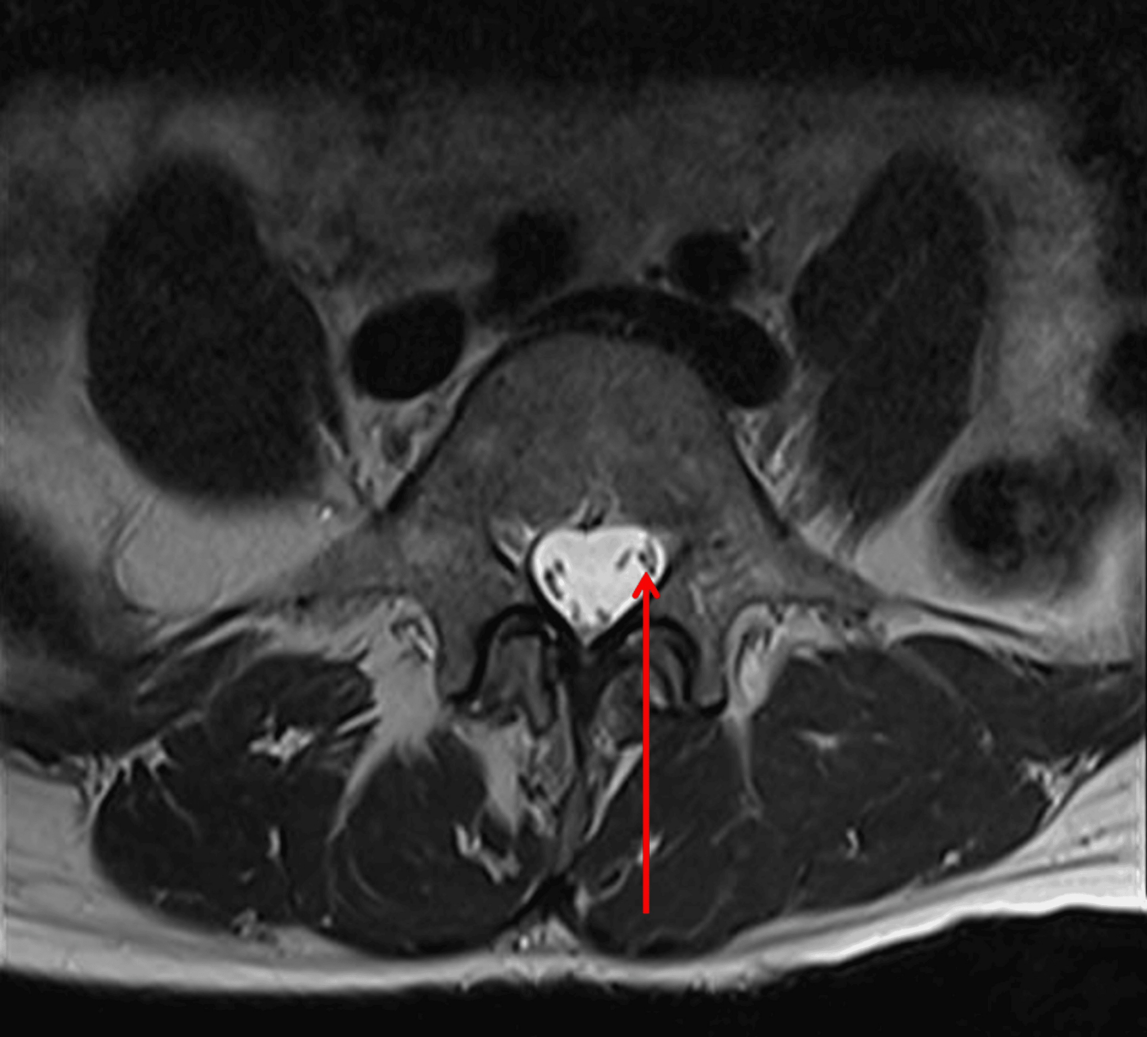
Axial T2-weighted MRI of the lumbar spine at L5. The red arrow depicts the duplicate exiting L5 nerve root.

The patient presented in this case demonstrated a duplicate left L5 exiting nerve root, which can be visualized using MRI in Figures 1-2. This anomaly is described as a type 1a NRA using the Neidre and McNab classification system. The conjoined roots are intradural and described as two nerve roots exiting a common sheath. This finding was evaluated intraoperatively and was confirmed upon secondary review of the sagittal MRI films.

Once this NRA was identified, several steps were taken to minimize the risk of iatrogenic injury. Such considerations included the use of a surgical microscope, secondary intraoperative review of left parasagittal films (Figure 1), meticulous skeletonization of neural elements from surrounding structures, adequate irrigation, prompt hemostasis, use of cottonoid patties to protect the underlying neural elements during removal of disc fragments, and light traction on the exiting roots.

In this particular case, careful review and tracking of the course of individual traversing and exiting nerve roots through each MRI slice may have prevented this near-miss event. Improved awareness and familiarity with the review of such findings may have increased the chances that this finding was not missed in the official radiological interpretation. Had the operating surgeon not been familiar with identifying such anomalies on imaging through decades of experience, it is possible that this patient may have sustained lifelong preventable paralysis. It is therefore critical for radiologists and surgeons alike to incorporate a system in their imaging review process that focuses on routinely identifying CNRs preoperatively.

## Conclusions

In the setting of this case study, a surgeon's preoperative awareness of NRAs and CNRs, use of careful dissection, and improved intraoperative visualization of neural structures are paramount to minimize the risks of surgical complications. Undiagnosed CRNs may not be discovered until operation and can result in increased risk of iatrogenic nerve root injury during routine spine surgery and other interventions. This case underscores the importance of systematic preoperative imaging review with specific attention to neural anatomy, particularly when standard MRI sequences may inadequately visualize subtle anatomical variants.

Further investigation to improve our capacity to distinguish CNRs preoperatively in patients with symptomatic lumbar disc herniation may decrease surgical risk. Additionally, improved didactic education on this topic and structured discussions during residency training for both radiologists and surgeons are essential to enhance recognition and management of these rare but clinically significant anomalies.
